# Cadmium exposure induces changes in gut microbial composition and metabolic function in long‐tailed dwarf hamsters, *Cricetulus longicaudatus*

**DOI:** 10.1002/ece3.11682

**Published:** 2024-07-04

**Authors:** Mengfan Tao, Kanglin Cao, Xinsheng Pu, Yu Hou, Lei He, Wei Liu, Yue Ren, Xin'gen Yang

**Affiliations:** ^1^ Shanxi Key Laboratory of Integrated Pest Management in Agriculture, College of Plant Protection Shanxi Agricultural University Taiyuan China; ^2^ Shanxi Forestry and Grassland General Engineering Station Taiyuan China

**Keywords:** cadmium exposure, *Cricetulus longicaudatus*, gut microbiota, metabolic function

## Abstract

Numerous studies have demonstrated that exposure to cadmium disrupts the diversity and composition of the gut microbiota, resulting in damage to organ tissue. However, there remains a lack of comprehensive understanding regarding the broader ecological reality associated with this phenomenon. In this study, we conducted a thorough evaluation of the effects of different concentrations of Cd (6, 12, 24, and 48 mg/L) over a period of 35 consecutive days on the organ viscera and the gut microbiota of long‐tailed dwarf hamsters, *Cricetulus longicaudatus* (Rodentia: Cricetidae), using histopathological analysis, 16S rDNA, and metagenome sequencing. Our findings revealed that the results suggest that Cd exposure induced liver, spleen, and kidney damage, potentially leading to increased intestinal permeability and inflammation. These alterations were accompanied by significant perturbations in the gut microbiota composition, particularly affecting potentially pathogenic bacteria such as Prevotella and Treponema within the gut ecosystem. Consequently, host susceptibility to underlying diseases was heightened due to these changes. Notably though, Cd exposure did not significantly impact the overall structure of the gut microbiota itself. Additionally, Cd exposure induced significant changes in the metabolic functions, with the pathways related to disease and environmental information processing notably enhanced, possibly indicating stronger innate defense mechanisms against external injuries among wild mammals exposed to Cd. This study offers a novel approach to comprehensively evaluate the significant impact of Cd pollution on ecosystems by investigating both structural and functional alterations in the digestive system, as well as disruptions in intestinal flora among wild mammals.

## Graphical abstract

This study can be used as a new approach to comprehend the stress response of wild mammals to Cd exposure and to further assess the significant impact of Cd pollution on ecosystems by investigating the structural and functional changes in their digestive system and the disruption of gut microbiota.

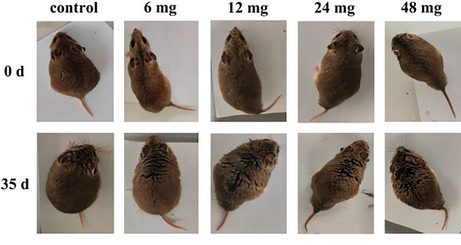

## 1 INTRODUCTION

The severity of heavy metal environmental pollution has escalated in recent years. While certain heavy metals serve as essential trace elements, the majority of them exhibit toxicity to all forms of life at elevated concentrations due to the formation of complex compounds within cells (Mohammed et al., 2011). Cadmium (Cd), a non‐essential heavy metal predominantly present as Cd^2+^ in the ecological environment, represents one of the most prevalent toxic metal pollutants found in agricultural soils and drinking water sources (He et al., 2019). Both humans and wild animals, including rodents, primates, and other mammals, have the potential to accumulate Cd in their bodies through dietary intake, resulting in a variety of detrimental health effects (Breton, Le Clère, et al., 2013; Jacob et al., 2018). Studies have demonstrated that, following oral ingestion, Cd^2+^ are transported by erythrocytes and subsequently penetrate into the host's bloodstream, subsequently accumulating accumulate in the kidney, intestine, and liver, leading to organ damage (Satarug, 2018; Tinkov et al., 2018). Among them, the intestine serves as the primary barrier following Cd intake (Nordberg & Nogawa, 2011). Exposure to Cd can induce inflammation, resulting in intestinal barrier impairment and disruption of both structure and metabolic function of gut microbiota (Breton, Le Clère, et al., 2013; Breton, Massart, et al., 2013; Zhao, Hyun, et al., 2006). Moreover, Cd exposure exerts adverse effects on the endocrine system, cardiovascular system, and reproductive system of both humans and wild animals via modulation of the immune system and microbial metabolites, thereby inducing diverse detrimental phenotypes and exerting subsequent influences on the gut microbiota (Jin et al., 2015, 2017; Zhang et al., 2015).

Following extensive adaptive evolution, the mammalian intestines have developed a diverse and intricate microbiota, establishing an homeostatic system (Bäckhed et al., 2004). As a metabolic “organ,” the gut microbiota plays a pivotal role in providing diverse metabolites and nutrients to the host, exerting influence on the host's immune function to combat pathogens and toxic compounds (Lavrinienko et al., 2021; Parfrey et al., 2018), and actively participating in the regulation of various physiological processes crucial for maintaining host health (Jin et al., 2017; Nayak, 2010). The alterations in the gut microbiota in response to environmental changes have been demonstrated to confer benefits to their hosts, which might yield enhanced capabilities to acclimate and adapt to the novel conditions. Therefore, measuring the impact of gut microbiota variation on the hosts' capacity to acclimate and adapt to novel environmental conditions will be necessary to assess the transcendence of gut microorganisms in the evolution of hosts (Alberdi et al., 2016). Rodents, which are widely distributed in the natural environment, the own characteristics, and external environment, can influence the structure and composition of host–gut microbiota (Suzuki, 2017). The inevitable extensive exposure to heavy metal pollutants can disrupt the diversity and composition of the gut microbiota, resulting in a significant decrease in microbial richness, which may profoundly impede associated gene pathways (He et al., 2020; Li et al., 2019; Liu et al., 2023). The study has demonstrated that Cd exposure significantly enhanced the relative abundances of Alistipes and Odoribacter in mice, while inducing notable reductions in Mollicutes and unclassified Ruminococcaceae, but no discernible alteration in microbial diversity (Zhai et al., 2017). However, investigations into the effects of both short‐ and long‐term Cd exposure have primarily focused on laboratory animals (e.g., *Rattus norvegicus* and *Mus musculus*), thus lacking a comprehensive ecological reality. In certain wild small mammals, species identity is thought to dominate over environment in shaping gut microbiota (Knowles et al., 2019; Suzuki, 2017). However, in numerous taxa (e.g., rodents, primates), the strength of host species effects varies depending on host ecology characteristics (Grond et al., 2020; Knowles et al., 2019). Therefore, the existence of a species‐specific impact of wild rodents cohabiting with humans in Cd‐polluted environments and the specificity of their gut microbiota response remain uncertain.

The long‐tailed dwarf hamster *Cricetulus longicaudatus* (Rodentia: Cricetidae) is primarily distributed in eastern and midwestern regions of China (Poplavskaya et al., 2018). In many regions of North China, the proportion exceed 70%, establishing it as the predominant species in agricultural land within this geographical area (Yang et al., 2022). As a typical heavy industrial region in China, the water and soil in North China suffer from severe contamination by heavy metals owing to frequent smelting activities and inadequate wastewater treatment. The Qinghe River basin in Shanxi Province has been found to have a serious accumulation of Cd, with concentrations reaching 12.46 mg/kg in certain areas, surpassing the threshold for moderate ecological risk intensity (Fu et al., 2013). However, the impact of Cd pollution on physiological damage and gut microbiota in *C. longicaudatus*, a representative wild animal in this region, remains uncertain, necessitating further investigation. In this study, a 35‐day experiment was conducted to examine the effects of Cd exposure on *C. longicaudatus*. HE staining, 16S rDNA sequencing, and metagenomic sequencing were employed to investigate tissue damage in organs, as well as alterations in gut microbiota structure and function following Cd exposure. The results of this study enhance our comprehension of the response exhibited by gut microbiota to Cd contamination in wild rodents and further elucidate the resilience of these animals toward stress caused by heavy metal pollution.

## 2 MATERIALS AND METHODS

### 2.1 Animals and experimental design

Healthy adult male *C. longicaudatus* were captured from Wutai County (113.3° E, 38.7° N), Lishi District (110.6° E, 37.3° N), Yuci District (112.5° E, 37.4° N), Xi County (110.6° E, 36.4° N), and Zuoquan County (113.6° E, 37.1° N) in Shanxi Province, North China, in September 2022. The captured target animals were transported to the laboratory and housed individually in plastic cages (33 × 21.5 × 16 cm) lined with wood chips. All cages were maintained under the ambient conditions of temperature at 23 ± 2°C, relative humidity at 55 ± 2%, and a natural light cycle. Water and food were provided ad libitum using standard rat chow (20.3% crude protein, 2.0% crude fiber, 5.2% crude fat, and 72.5% water and others). All animals were kept under these conditions for 60 days, after which the first fecal sampling was performed. The animals were divided into one control and four treatment groups (five samples per group) and treated for 35 days, during which the foods were provided with standard rat chow. For the control group, the samples were given pure water. For the treatment groups, the samples were fed CdCl_2_ (in pure water, sourced from Shanghai Macklin Biochemical Co., Ltd, 99.99% pure) to mimic environmental Cd exposure at doses of 6, 12, 24, and 48 mg/L, respectively (Fu et al., 2013). Fecal sampling and weight measurement for each individual were performed every 7 days, followed by immediate storage at −80°C refrigerator for subsequent analysis. The phenotypic dynamics of *C. longicaudatus* during exposure to Cd were observed. After the last fecal sampling, the hamsters were stunned with CO_2_ before being killed by neck dislocation, and the liver, spleen, kidney, and ileum tissues were immediately collected and fixed for preservation. This animal experiment was approved by the Animal Experiment Ethics Committee, Shanxi Agricultural University. All procedures were conducted according to the Regulations for the Administration of Laboratory Animals established by the Ministry of Science and Technology of the People's Republic of China (2017 Revision). After a 35‐day exposure to Cd, we collected a total of 150 fecal samples for high‐throughput sequencing of 16S rDNA, followed by random selection of three fecal samples from both the control group and each treatment group for metagenome sequencing.

### 2.2 Histopathological analyses

The preserved tissues underwent histopathological analysis. They were fixed in 4% paraformaldehyde and subsequently were adequately fixed before being embedded in paraffin and sectioned into 5‐μm sections. The paraffin sections were immersed sequentially in environmental friendly dewaxing transparent liquid I for 20 min, environmental friendly dewaxing transparent liquid II for 20 min, anhydrous ethanol I for 5 min, anhydrous ethanol II for 5 min, and 75% ethanol for 5 min and then rinsed with tap water. Before staining, the sections were treated with the high‐definition constant staining pretreatment solution for 1 min. Subsequently, they were immersed in the hematoxylin solution for 3–5 min and then rinsed with tap water. Following this, the sections were treated with hematoxylin differentiation solution and rinsed with tap water, then were treated with hematoxylin bluing solution and rinsed with tap water. After that, the sections were immersed in 95% ethanol for 1 min and stained with eosin for 15 s anhydrous ethanol I, anhydrous ethanol II, and anhydrous ethanol III for 2 min each. This was followed by immersion in normal butanol I and normal butanol II for 2 min each, as well as xylene I and xylene II for 2 min each. Finally, the neutral gum was applied to seal the sections before microscope inspection, image acquisition, and analysis.

### 2.3 16S rDNA sequencing of gut microbiota and data analyses

Total DNA from fecal samples were extracted with E.Z.N.A™ Magbind Soil DNA Kit (Omega, M5635‐02, USA), following the manufacturer's instructions. We measured the concentration of the DNA using a Qubit 4.0 (Thermo, USA) to ensure that adequate amounts of high‐quality genomic DNA had been extracted. Two universal bacterial 16S rDNA gene amplicon PCR primers (PAGE purified) were used: the amplicon PCR forward primer (5′‐CCTACGGGNGGCWGCAG‐3′) and amplicon PCR reverse primer (5′‐GACTACHVGGGTATCTAATCC‐3′) (designed and synthesized by Sangon BioTech, Shanghai). The reaction was set up as follows: microbial DNA (10 ng/μL) 2 μL; amplicon PCR forward primer (10 μM) 1 μL; amplicon PCR reverse primer (10 μM) 1 μL; and 2× Hieff® Robust PCR Master Mix (Yeasen, 10105ES03, China) (total 30 μL). The plate was sealed and PCR performed in a thermal instrument (Applied Biosystems 9700, USA) using the following program: one cycle of denaturing at 95°C for 3 min, first five cycles of denaturing at 95°C for 30 s, annealing at 45°C for 30 s, elongation at 72°C for 30 s, then 20 cycles of denaturing at 95°C for 30 s, annealing at 55°C for 30 s, elongation at 72°C for 30 s, and a final extension at 72°C for 5 min. The PCR products were checked using electrophoresis in 2% (w/v) agarose gels in TBE buffer (Tris, boric acid, EDTA) stained with ethidium bromide (EB) and visualized under UV light. We used Hieff NGS™ DNA Selection Beads (Yeasen, 10105ES03, China) to purify the free primers and primer dimer species in the amplicon product. Samples were delivered to Sangon BioTech, Shanghai, for library construction using universal Illumina adaptor and index using the Illumina MiSeq system (Illumina MiSeq, USA) (Caporaso et al., 2012), according to the manufacturer's instructions. After sequencing, the two short Illumina readings were assembled by PEAR software (version 0.9.8) (Zhang et al., 2014) according to the overlap and fastq files were processed to generate individual fasta and qual files, which could then be analyzed by standard methods (Schmieder & Edwards, 2011). The effective tags were clustered into operational taxonomic units (OTUs) and sequences were sorted into each sample based on the OTUs. The tag sequence with the ≥97% similarity using Usearch software (version 11.0.667). Chimeric sequences and singleton OTUs (with only one read) were removed, after which the remaining highest abundance was selected as a representative sequence within each cluster. Bacterial and fungal OTU representative sequences were classified taxonomically by blasting against the RDP Database and UNITE fungal ITS Database, respectively (Edgar, 2010).

The α‐diversity indices (Chao1 indices) were quantified in terms of OTU richness. To assess sample adequacy, rarefaction curves of the observed numbers of OTUs were constructed, all α diversity indices were calculated with Mothur software (version 3.8.31). The OTU rarefaction curve and rank abundance curves were plotted in R (version 3.6.0) (Edgar, 2013). To estimate the diversity of the microbial community of the sample, multiple group comparisons were made using analysis of variance (ANOVA) test. Beta diversity assesses the differences in the microbiome among distinct groups and over different time points within the same group, and generally constrains principal component analysis (PCA) dimensionality reduction methods to obtain visual representation. These analyses were visualized using R vegan package (version 2.5‐6), and finally, the inter‐sample distances were presented as scatterplots. Difference comparison is used to identify features with significantly different abundances between groups using STAMP (version 2.1.3) (Parks et al., 2014) and LEfSe (version 1.1.0) (Segata et al., 2011). Correlation coefficients and *p*‐values between communities/OTUs were calculated using SparCC (version 1.1.0) (Friedman & Alm, 2012), and correlation matrix heatmaps were drawn using R corrplot package (version 0.84). R ggraph package (version 2.0.0) (Bartley et al., 2015) is used to build network graphs.

### 2.4 Metagenome sequencing and data analyses of gut microbiota

The total amount of DNA in each sample was 500 ng as input material for DNA sample preparation. Sequencing libraries were generated using Hieff NGS® MaxUp II DNA Library Prep Kit for Illumina® (12200ES96, YEASEN, China) following manufacturer's instructions, and index codes were added to attribute sequences to each sample. In a nutshell, DNA was broken into fragments of about 500 bp using Covaris 220. In order to purify DNA fragments of 500 bp, the library fragments were purified with Hieff NGS™ DNA Selection Beads DNA (12601ES56, YEASEN, China). The purified DNA was end repair, adapter ligation, and fragment selection. Then, PCR was performed with 2× Super Canace®II High‐Fidelity Mix, primer mix (p5/p7), and adaptor‐ligated DNA. At last, PCR products were purified (Hieff NGS™ DNA Selection Beads) and library quality was assessed on the Qubit®4.0 Fluorometer. The libraries were then quantified and pooled. Paired‐end sequencing of the library was performed on the NovaSeq 6000 sequencers (Illumina, USA).

Fastp (version 0.36) was used for evaluating the quality of sequenced data (Bolger et al., 2014). Raw reads were filtered according to several steps: (1) removing adaptor sequence; (2) removing low‐quality bases from reads 3′ to 5′ (*Q* < 20), using a sliding window method to remove the base value less than 20 of reads tail (window size is 4 bp); (3) finding overlap of each pair of reads and properly correcting inconsistent bases within the interval; (4) removing reads with reads length less than 35 nt and its pairing reads. The remaining clean data were used for further analysis.

Megahit (version 1.2.9) was used to perform multi‐sample mixed splicing to obtain preliminary spliced contig sequences (Li et al., 2015). Clean reads were subsequently mapped back to the spliced results using bowite2 (version 2.1.0) to extract unmapped reads (Langmead & Salzberg, 2012) and spliced again using SPAdes (version 3.13) (Bankevich et al., 2012) to obtain low‐abundance contigs. MetaWRAP (version1.3.2) was used to perform a series of binning and processes, including bin sorting, bin purification, bin quantification, bin reassembly, and bin identification performed in sequence. After filtering, a draft genome of a single bacteria with high integrity and low contamination was obtained.

Prodigal (version 2.60) was used to predict the ORF of the splicing results, select genes with a length greater than or equal to 100 bp, and translate them into amino acid sequences (Hyatt et al., 2010). For the gene prediction results of each sample, the CD‐HIT (version 2.60) (Hyatt et al., 2010) was used for de‐redundancy to obtain a non‐redundant gene set. Salmon (version 1.5.0) was used to construct a specific index of non‐redundant gene sets, using a dual‐phase algorithm and a method of constructing a bias model to accurately quantify the abundance of genes in each sample, and calculate gene abundance based on gene length information.

DIAMOND (version 0.8.20) was used to compare the gene set with Kyoto Encyclopedia of Genes and Genomes (KEGG) and other databases to obtain species annotation information and functional annotation information of genes. Screening conditions were as follows: *E*‐value <1e−5, Score >60. Based on gene set abundance information and annotation information, species abundance and functional abundance were obtained, and multi‐directional statistical analyses such as species and functional composition analysis, species and functional difference analysis, and sample comparison analysis were performed.

### 2.5 Statistical analyses

The experimental results were expressed as mean ± standard deviation (mean ± SD). SPSS 26.0 software was used to calculate the relative abundance and Alpha diversity of samples among groups by repeated‐measures ANOVA. Statistical differences between treatments were considered significant at *p < .05, **p < .01, and ***p < .001.

## 3 RESULTS

### 3.1 Phenotypic analysis

**FIGURE 1:**
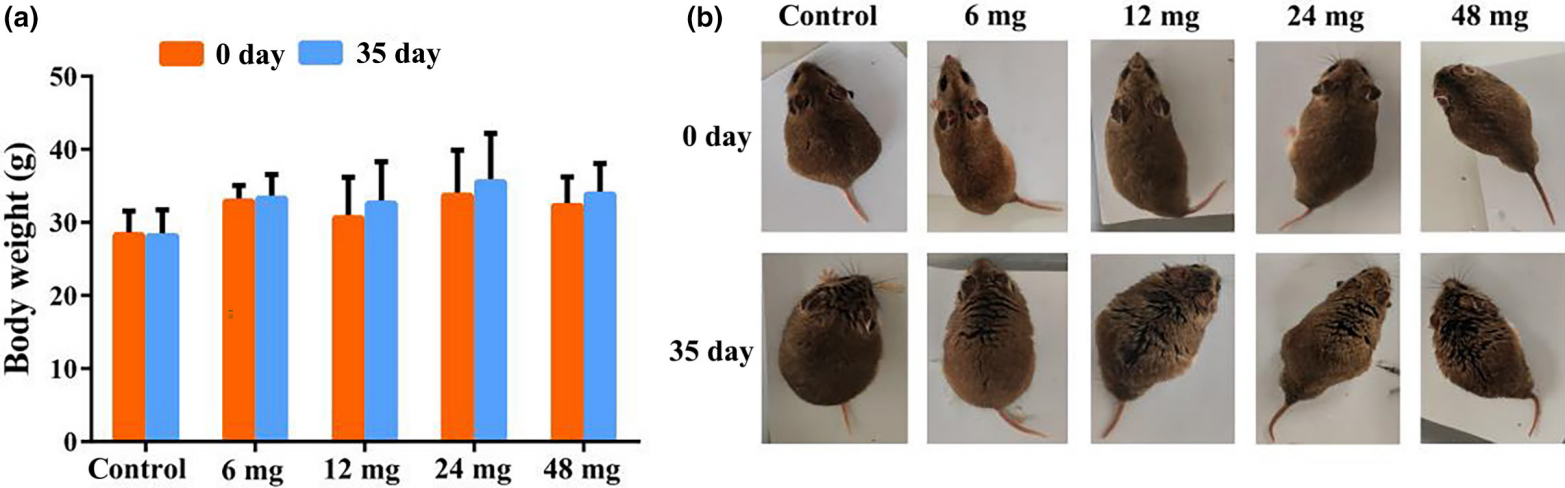
Effect of Cd exposure on the phenotype of *C. longicaudatus*. (a) Changes in body weight after Cd exposure in *C. longicaudatus*. (b) Phenotypic changes in *C. longicaudatus*.

After grouping, the body weight did not significantly among the experimental groups (*p* > .05). No significant changes in body weight were detected (*p* > .05), and no mortalities were recorded throughout the entire duration of Cd exposure (35 days) (Figure 1a). However, individuals in the treatment groups exhibited coarse hair texture and alopecia (Figure 1b).

### 3.2 Organ tissue damage caused by Cd exposure

**FIGURE 2:**
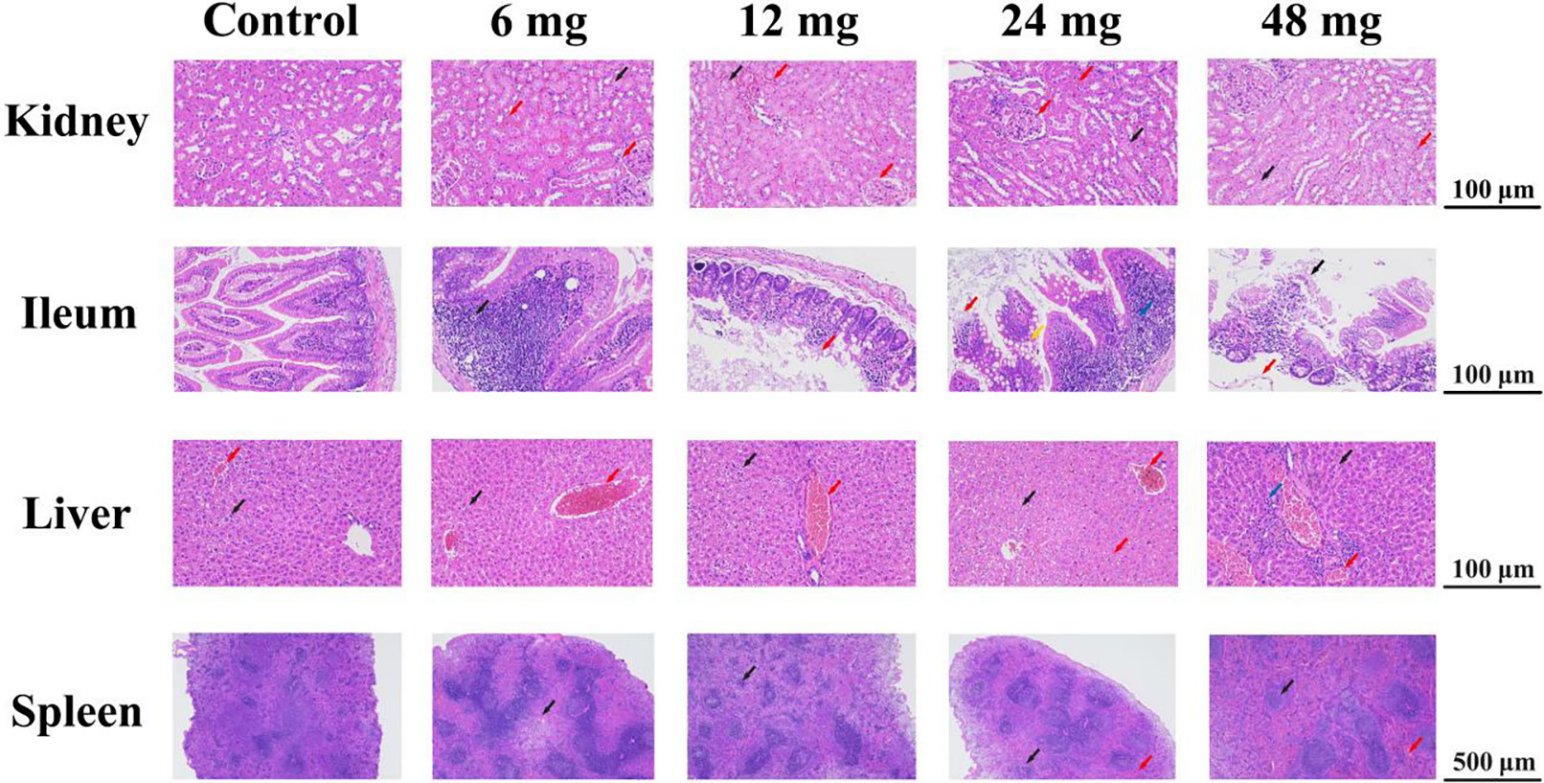
Organ tissue damage caused by Cd exposure. Tissue sections of kidney, ileum, liver, and spleen after Cd exposure were stained with H&E. Black arrows indicate organ cell degeneration and ileal wall damage, red arrows indicate potential inflammation and ileal villi shedding, yellow arrows indicate increased goblet cells in ileal tissue, and blue arrows indicate lymphocyte infiltration.

After a duration of 35 days, the kidney, ileum, liver, and spleen of individuals from each group were subjected to histological analysis. The microscopic examination revealed normal appearance of organ tissues in the control group (Figure 2). However, in the treatment groups, an increase in concentration of Cd led to an escalation in organ tissue damage (Figure 2). The individuals of the treatment groups exhibited focal degeneration of renal tubular epithelial cells at the medullary junction, as well as congestion of medullary, interstitial, and glomerular capillaries (Figure 2). In the ileum, a small area of intestinal gland necrosis and loss with shedding of intestinal villi were observed (Figure 2). These lesions became more obvious with increasing concentration (Figure 2). Blood vessel stasis and hepatocyte granule degeneration were also observed in the liver (Figure 2), while splenic sinus stasis dilatation was observed in both 24 and 48 mg groups (Figure 2).

### 3.3 Effects of Cd exposure on gut microbial diversity

**FIGURE 3:**
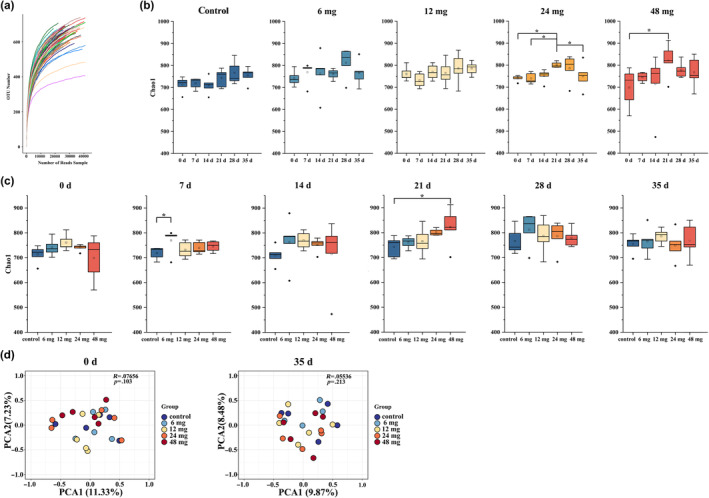
Effects of Cd exposure on gut microbial diversity *C. longicaudatus*. (a) Chao index for richness, *p < .05. (b) Principal coordinate analysis (PCA) of the gut microbial compositions of *C. longicaudatus* during the experimental period. (c) Rarefaction curves.

A total of 8,956,008 reads were obtained from 150 fecal samples of *C. longicaudatus* through 16S rDNA high‐throughput sequencing. Following read splicing, filtering, and demultiplexing the reads, a total of 8,833,992 valid sequences were acquired. These sequences were subsequently subjected to clustering analysis at a similarity threshold of 97%, resulting in the identification of a total of 1154 OTUs. This comprehensive coverage suggested that most of the microbes in the diversity analysis had been encompassed (Figure 3a).

The results of high‐throughput sequencing of 16S rDNA revealed an ecological imbalance in the gut microbiota of *C. longicaudatus* during Cd exposure in the treatment groups. The community richness index Chao1 of the 24 and 48 mg groups initially showed a significant increase, followed by a subsequent decline with increasing Cd exposure time (Figure 3b). On Day 7, the Chao1 index of 6 mg group was significantly higher than that of the control group (*p* = .023) (Figure 3b). By Day 21, there was a significant decrease in the Chao1 index for the 48 mg group compared to the control group (*p* = .018) (Figure 3c). The PCA revealed no statistically significant difference in the diversity of gut microbiota among groups on Day 35 (*p* = .213), suggesting that Cd exposure did not exert a discernible impact on microbial diversity during this particular time (Figure 3d).

### 3.4 Changes in Cd specificity in the gut microbiota

**FIGURE 4:**
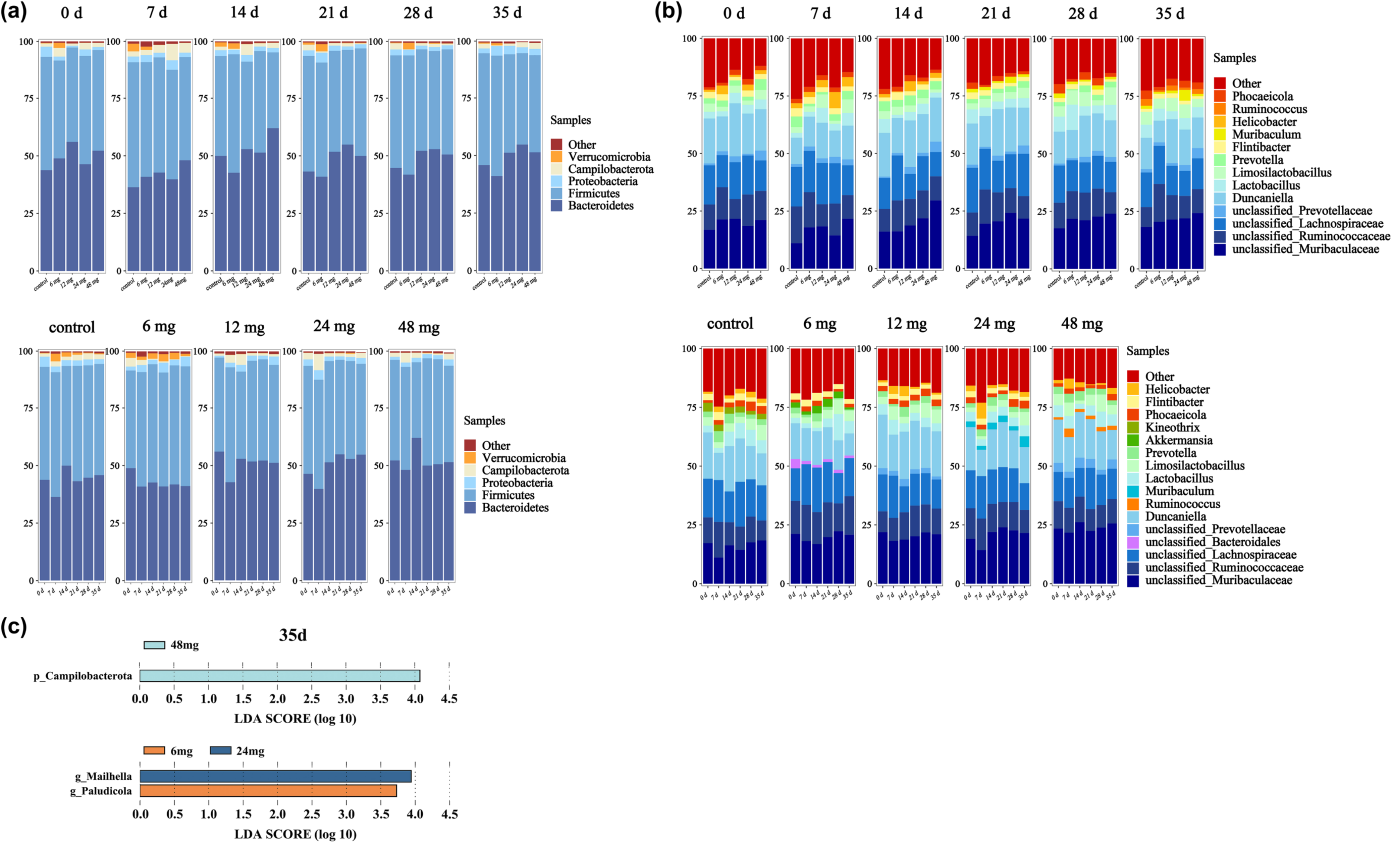
Changes in metal specificity in the gut microbiota of *C. longicaudatus* after Cd exposure. (a) Species distribution at the phylum level of gut microbiota in the *C. longicaudatus* population. (b) Species distribution at the genus level of gut microbiota in *C. longicaudatus* populations. (c) Differential species of gut microbiota in *C. longicaudatus* under Cd exposure at the phylum and genus levels on Day 35.

Significant alterations were observed in the abundance of certain microbiota in the gut of *C. longicaudatus* during Cd exposure, spanning from phylum to genus level. At the phylum level, Bacteroidetes and Firmicutes emerged as predominant constituents within the gut microbiota. The relative abundances of Firmicutes (*p* = .035), Proteobacteria (*p* = .016), Campylobacter (*p* = .011), and Verrucomicrobia (*p* = .045) exhibited significant decreases with increasing Cd exposure dose, in comparison with the control group (Figure 4a). In contrast, there was a significant increase in the relative abundance of Bacteroidetes (*p* = .024) (Figure 4a). In the temporal dimension, the relative abundance of Proteobacteria exhibited a significant increase with prolonged exposure to Cd in the 12 mg group (Figure 4a). The relative abundances of Proteobacteria (*p* = .048), Campilobacterota (*p* = .032), and Verrucomicrobia (*p* = .038) showed significant decreases in the 24 mg group (Figure 4a). Conversely, a significant increase in the relative abundance of Bacteroidetes was observed (*p* = .018) (Figure 4a). Furthermore, the relative abundance of Campilobacterota showed a significant decrease in the 48 mg group (*p* = .006) (Figure 4a). The LEfSe analysis revealed a significant enrichment of Campilobacterota in the 48 mg group at 35 days (Figure 4c).

The predominant taxa of the gut microbiota in *C. longicaudatus* at the generic level comprised Duncaniella, unclassified_Lachnospriaceae, unclassified_Ruminococcaceae, and unclassified_Muribaculaceae. Their relative abundances were significantly influenced by Cd exposure, with the exception of unclassified_Lachnospriaceae (Figure 4b). The relative abundances of Duncaniella (*p* = .041) and unclassified_Muribaculaceaeun (*p* = .042) exhibited significant increases with increasing exposure dose of Cd, while the relative abundance of unclassified_Ruminococcaceae decreased significantly (*p* = .024) (Figure 4b). After increasing the exposure time to Cd, significant increases in the relative abundance of Lactobacillus (*p* < .001) and Limosilactobacillus (*p* = .012) were observed in the 12 mg group, while these of Duncaniella (*p* = .036) and Phocaeicola (*p* = .018) decreased significantly (Figure 4b). The relative abundances of Duncaniella (*p* = .037) and unclassified_Muribaculaceae (*p* = .015) significantly increased in 24 mg group (Figure 4b). In the 48 mg group, there was a significant increase in the relative abundance of Limosilactobacillus (*p* = .038), while the relative abundances of Duncaniella (*p* = .027) and Lactobacillus (*p* = .044) decreased significantly (Figure 4b). Additionally, on 21 and 28 day, the relative abundances of Muribaculum (*p* = .047) and Prevotella (*p* = .025) increased, while that of Helicobacter decreased (Figure 4b). At 35 day, Ruminococcus showed a significant, increase in abundance, while Muribaculum (*p* = .018) and Prevotella (*p* = .029) exhibited significant decreases (Figure 4b). The LEfSe analysis showed a significant enrichment of Mailhella and Paludicola in the 12 and 24 mg groups at 35 days (Figure 4c).

### 3.5 Cd concentration differences in the symbiotic network of gut microbiota

**FIGURE 5:**
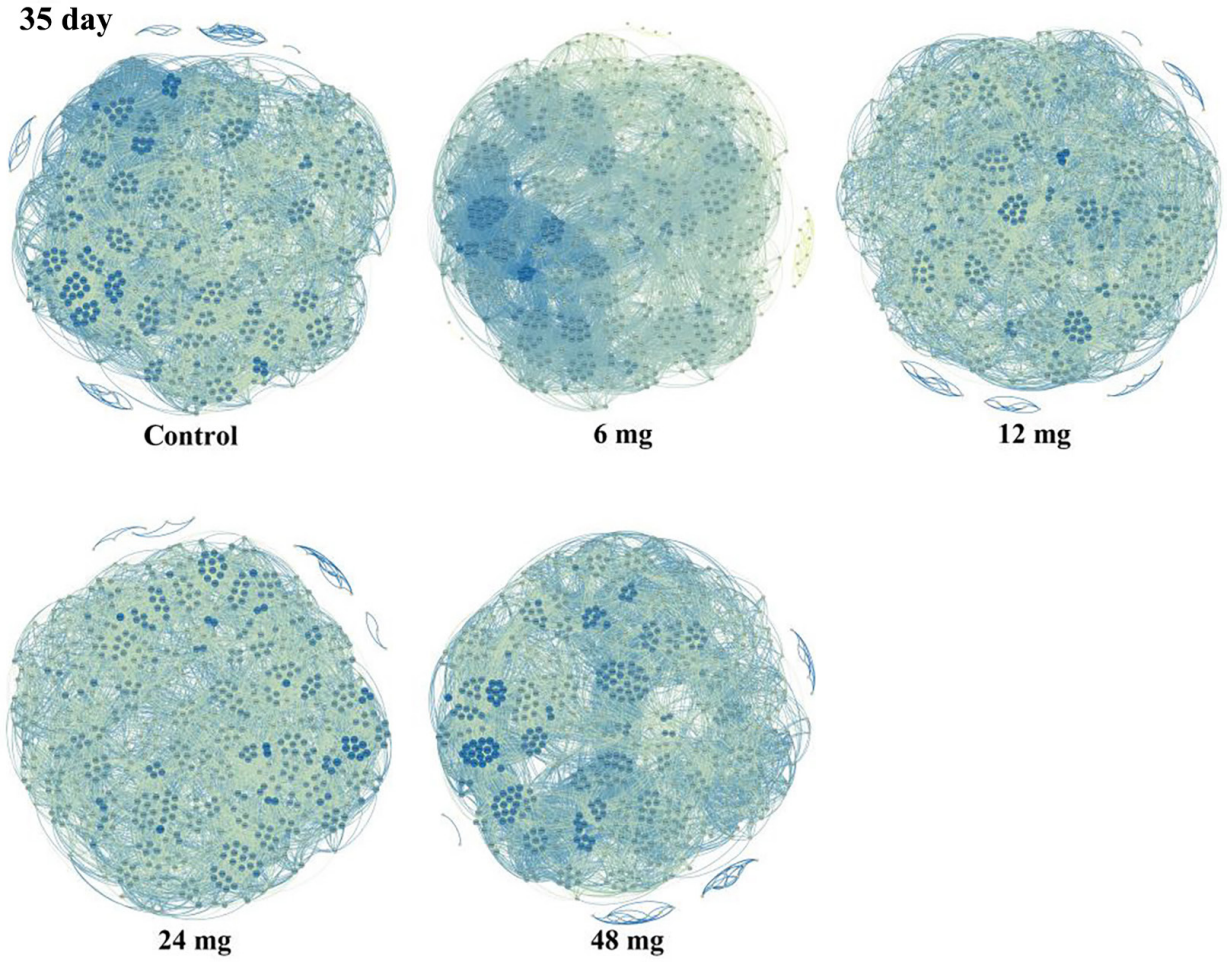
Cd Concentration differences in the symbiotic network of gut microbiota in *C. longicaudatus*. Symbiotic networks of the top 600 operational taxonomic units (OTUs) among different group nodes. Nodes represent OTUs and their sizes indicate different relative abundance. Links between the nodes indicate a significant correlation between 2 OTUs (blue: positive; yellow: negative).

The symbiotic network was analyzed on the 35th day, revealing that the gut microbiota of *C. longicaudatus* exhibited the highest complexity in the 48 mg group, with a selection of 600 nodes in each group (Figure 5). In the control group, there were 11,381 links exhibiting a dominant positive correlation of 63.95%. The 6 mg group displayed 11,771 links with a positive correlation of 59.13%, while the 12 mg group exhibited 11,211 links with a positive correlation of 60.80%, and the 24 mg group demonstrated a total of 11,058 links with a positive correlation rate of 53.37%. Remarkably, the high‐dose (48 mg) group presented an impressive total count of 11,853 links and shown an elevated positive correlation rate which reaching 68.94% (Figure 5). Compared to the control group, the 6, 12, and 24 mg groups exhibited a decrease both in the overall number of links and positive correlations, while an increase was observed in the 48 mg group. Furthermore, significant differences in network structure between the control and four treatment groups were also evident for various important network topological properties such as average degree, average weight, and density (Table 1).

**TABLE 1:** Network topology table on 35 days.

Group	Node couns	Edge couns	Positive edges (%)	Negative edges (%)	Average degree	Density	Clustering coefficient	Average Path length	Diameter	Modularity
Control	600	11,381	63.95	36.05	11.593	0.063	0.576	3.165	5	1.637
6 mg	600	11,771	59.13	40.87	8.348	0.066	0.573	3.171	6	2.178
12 mg	600	11,211	60.80	39.20	37.37	0.062	0.574	3.128	5	2.205
24 mg	600	11,058	53.37	46.63	3.073	0.062	0.579	3.157	6	5.55
48 mg	600	11,853	68.94	31.06	16.257	0.066	0.580	3.180	6	1.31

### 3.6 Analysis of potential pathogenic bacteria in the gut

**FIGURE 6:**
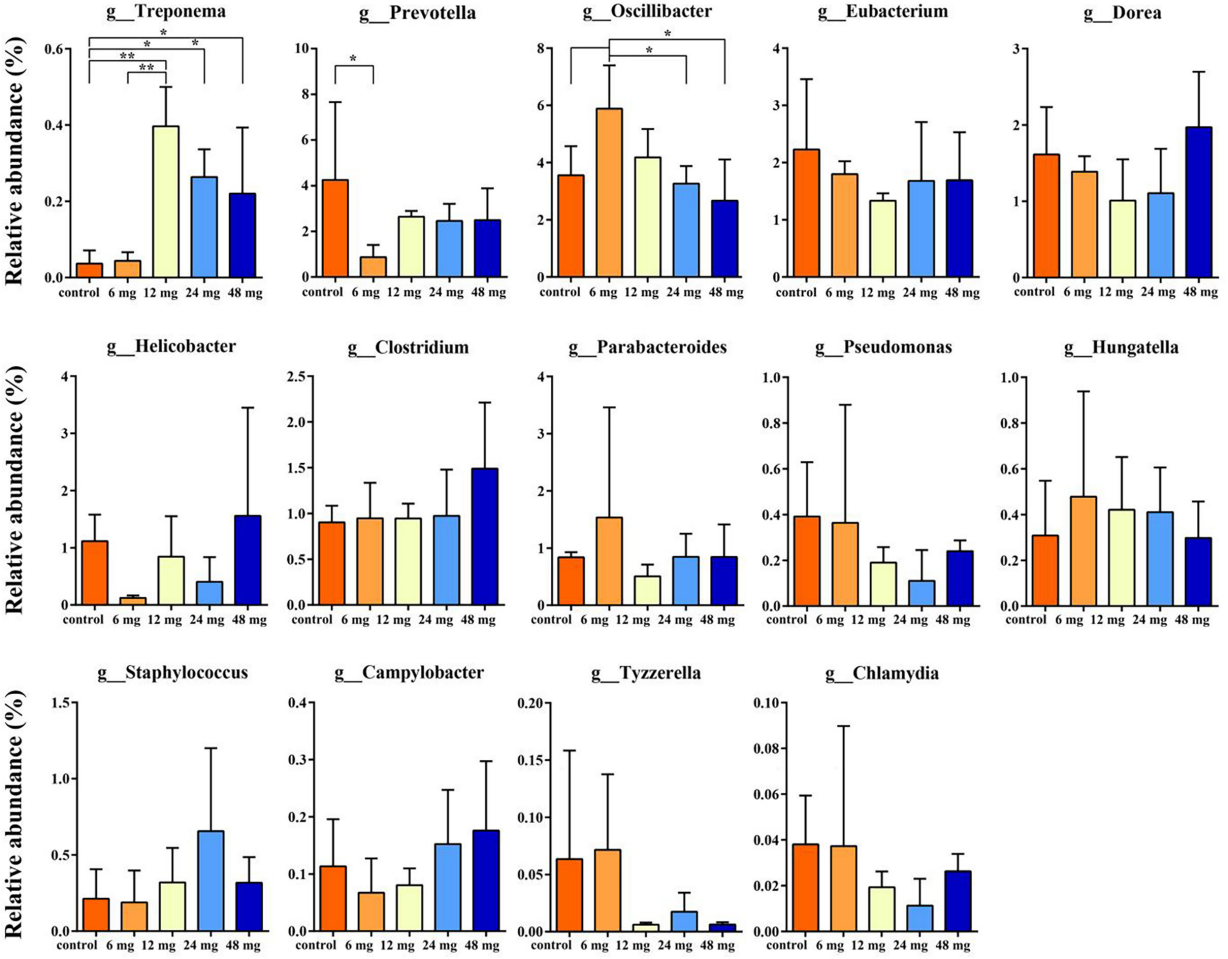
Analysis of potential pathogenic bacteria in the intestinal tract of *C. longicaudatus*. *p < .05, ***p < .01.

A total of 827,227,072 valid sequences and 5,075,111 contigs were obtained from 15 fecal samples of *C. longicaudatus* through metagenomic sequencing. At the generic level, analysis of potential pathogens for the top 10% of the gut microbiota revealed 14 previously reported pathogens, which were designated as potential pathogens for this study, including Treponema (Sela, 2001), Prevotella (Nakajima et al., 2020), Oscillibacter (Elwell et al., 2016), Eubacterium (Ley, 2016), Dreo (Konikoff & Gophna, 2016), Helicobacter (Mukherjee et al., 2020), Clostridium (Chaput et al., 2017), Parabacteroides (Zaragoza et al., 2019), Pseudomonas (Ezeji et al., 2021), Hungatella (Peix et al., 2009), Staphylococcus (Demir Çuha et al., 2021), Campylobacter (Yang et al., 2021), Tyzzerella (Heaton et al., 2020), and Chlamydia (Bulajić et al., 2022) (Figure 6). The difference analysis revealed a significant increase in Treponema compared to the control group (*p* = .001), whereas Prevotella (*p* = .035) and Oscillibacter (*p* = .007) exhibited significant decreases (Figure 6). Other potential pathogens did not show significant changes (Figure 6).

### 3.7 Effects of Cd exposure on metabolic function of gut microbiota

**FIGURE 7:**
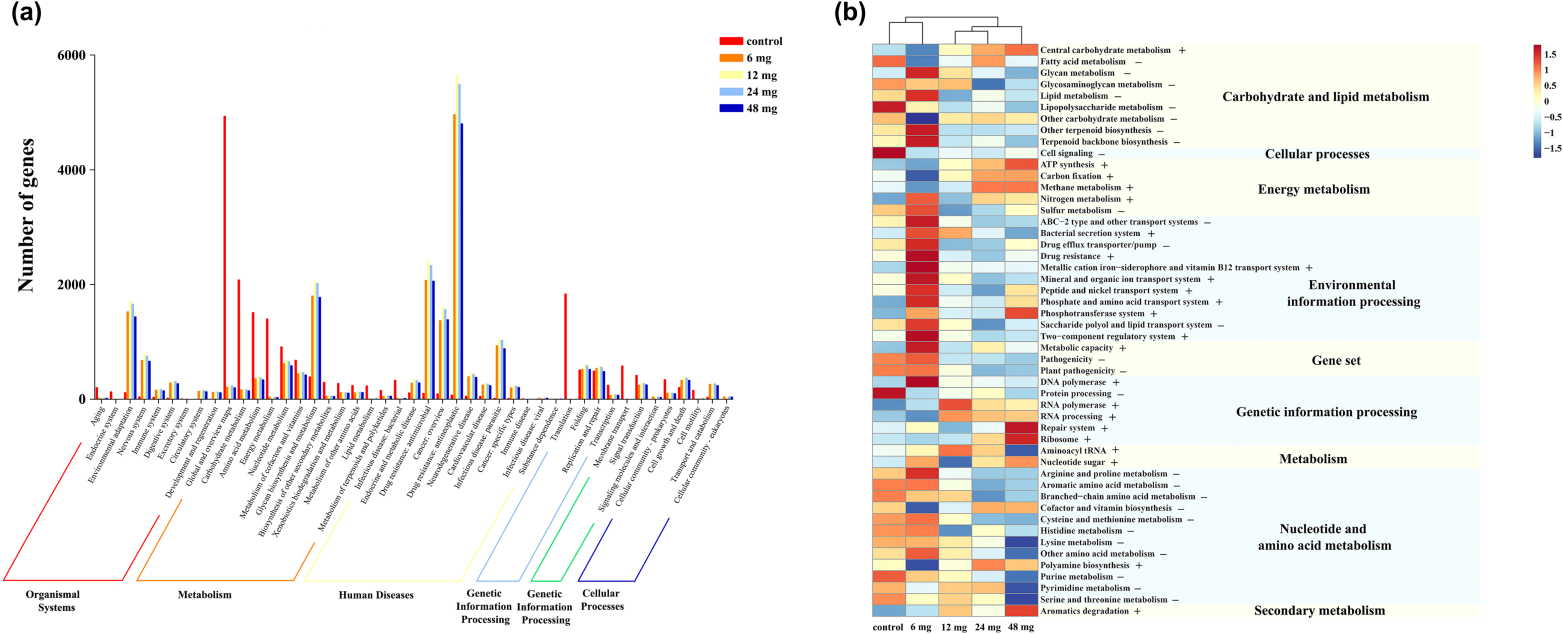
Effects of Cd exposure on metabolic function of gut microbiota in *C. longicaudatus*. (a) Histogram of KEGG pathway class one and class two statistics. (b) Heat map of KEGG pathway class three annotation.

DIAMOND was used to compare the protein sequence of the gene set with the KEGG database in order to obtain the corresponding KO number for the sequence. The Pathway and Module annotation information of the sequence was obtained based on the connection between KO and Pathway and Module. The altered metabolic pathways included amino acid metabolism, carbohydrate metabolism, energy metabolism, other biosynthesis and metabolism processes, lipid metabolism, sugar biosynthesis, and metabolism processes, as well as pathways related to genetic information processing, environmental information processing, and diseases. Among different metabolic functions, more than half of the metabolic functions in the Cd‐treated groups were significantly decreased compared with the control group, including essential metabolic pathways such as biosynthesis and degradation of cysteine, methionine, and lysine, as well as metabolic functions related to carbohydrate and lipid metabolism like fatty acid metabolism, lipid metabolism, and glycosaminoglycan metabolism. Notably, the pathways related to disease and environmental information processing showed significant increases, particularly in the repair system and phosphotransferase system (Figure 7).

## 4 DISCUSSION

Oral ingestion of Cd‐containing food or water is the main way for animals to suffer from Cd hazards in the living environment. Despite extensive investigations into the toxicity of Cd on various model animals, primarily focusing on Cd exposure‐induced intestinal damage and organ‐specific accumulation in *R. norvegicus*, *M. musculus*, etc. (Li et al., 2019; Liu et al., 2023; Wong et al., 1980), there remains a dearth of comprehensive research on the impact of this heavy metal on wild mammals. Previous studies have demonstrated that the gut serves as the primary barrier following oral ingestion of Cd, and organs directly exposed to Cd, which play crucial roles in nutrient metabolism and energy acquisition, ultimately resulting in weight loss (He et al., 2020). In the present study, it was observed that the intestines exhibited the most severe organ injuries. Distorted ileum tissue, extensive villi shedding and melting, and an increase in goblet cells were particularly prominent (Figure 2). With an increase in Cd exposure dose, the species exhibited more pronounced cell degeneration and congestion symptoms in the liver, spleen, and kidney. However, no abnormalities were detected in organ and tissue morphology. These pathological changes ultimately resulted in a coarse hair texture and alopecia (Figure 1b). However, there was no significant alteration in body weight (Figure 1a), which contradicts previous findings (Zhai et al., 2017) and may suggest that *C. longicaudatus* exhibits greater resilience and adaptability to adversity compared to laboratory mice or rats.

The species identity is believed to play a crucial role in shaping the gut microbiota of wild rodents, but only a few studies have investigated the regulation and preservation of the gut microbiota under adverse conditions (Knowles et al., 2019; Suzuki, 2017). This study confirmed a significant disturbance in the Chao1 index of gut microbiota during Cd exposure (Figure 3b,c). However, no significant differences were observed in the gut microbiota structure between groups (Figure 3d). This may be attributed to the ability of Cd exposure to alter the abundance of certain bacterial strains in the rodent gut without significantly affecting the overall structure of the gut microbiome (Fazeli et al., 2011; Sadykov et al., 2009), thus providing further evidence supporting the strong resilience of the gut microbiota of *C. longicaudatus* against Cd exposure. Previous studies have highlighted a close association between intestinal injury and an ecological imbalance of the gut microbiota (Camilleri et al., 2012; Jakobsson et al., 2015). Our study revealed significant alterations in the richness of the gut microbiota in *C. longicaudatus*, indicating dysfunctionality of their microbial ecosystem, which further contribute to the occurrence of intestinal injury in the Cd‐treated groups.

At 35 days post‐exposure to Cd, comparing to the control group, the gut microbiota exhibited the most intricate collinear network among the group exposed to the 48 mg group of Cd, while other treatment groups displayed a comparatively simpler collinear network (Figure 5). Moreover, it was found that environmental stress significantly influenced the positive network cohesion and complexity (Fan et al., 2022) indicating a substantial impact of Cd exposure on the gut microbiota. Therefore, the 48 mg group may elicit a stress response in *C. longicaudatus* and cause their innate self‐protective mechanism under adverse conditions. Persistent alterations in gut microbiota were observed during 35 days of Cd exposure in the Cd‐treated group, while Bacteroidetes and Firmicutes remained the predominant phyla in the gut of *C. longicaudatus*, consistent with previous findings in mice (Zhai et al., 2017) (Figure 4a). Previous study has demonstrated the significant involvement of Campilobacterota in various diseases (Kaakoush et al., 2015). In this study, a notable decline in the abundance of Campilobacterota was observed in the high‐dose groups (24 and 48 mg), potentially indicating a stress response to Cd intake adversity in long‐tailed dwarf hamsters. The substantial enrichment of Campilobacterota in the 48 mg group on 35 d may suggest that excessive Cd consumption poses a potential disease risk. The results of this study indicate that certain beneficial bacteria also undergo changes, such as Lactobacillus. Lactobacillus plays a crucial role in maintaining intestinal health and enhancing immunity (Dempsey & Corr, 2022). The relative abundance of Lactobacillus significantly increases in the 12 mg group, while it decreases significantly in the 48 mg group. Lactobacillus are beneficial bacteria in the gut, and there have been reports of Lactobacillus providing benefit in infectious diarrhea (Slover & Danziger, 2008). This finding further emphasizes the pivotal regulatory function of gut microbiota in the physiological processes of *C. longicaudatus*. It is noteworthy that Duncaniella exhibited significant fluctuations throughout the experiment. However, its function remains elusive and requires further investigation. These perturbations in the gut microbiota equilibrium may potentially augment the intestinal absorption of this metal in gut microbiota, thereby exacerbating Cd toxicity.

Studies have demonstrated that Cd exposure can modulate the abundance of specific pathogens (Yang et al., 2021). The reduction of Prevotella in the gut is associated with the pathogenesis of certain diseases (Ley, 2016). Oscillibacter significantly decreases when inflammation occurs in the gut (Konikoff & Gophna, 2016), while Treponema is associated with periodontal diseases such as early‐onset periodontitis, necrotizing ulcerative gingivitis, and acute pericoronitis (Sela, 2001). Eubacterium plays a pivotal role in regulating intestinal inflammation, and its reduction may contribute to disease development (Mukherjee et al., 2020). Pseudomonas is associated with intestinal barrier dysfunction and infection (Peix et al., 2009). Dorea exhibits proinflammatory properties (Chaput et al., 2017). Cd exposure can decrease the abundance of Prevotella and Lachnoclostridium (Yang et al., 2021). We conducted an analysis on the gut microbiota of *C. longicaudatus* and observed a significant decrease in Prevotella and Oscillibacter, while Treponema exhibited a substantial increase. Additionally, 11 other pathogenic bacteria displayed a certain degree of disturbance (Figure 6), suggesting that *C. longicaudatus* may experience heightened susceptibility to potential diseases following Cd ingestion.

Many of the functions of the normal gut microbiome can be performed by multiple microbial groups (Human Microbiome Project Consortium, 2012), and merely knowing the taxonomic abundance of microbes cannot provide us with a complete understanding on their impact the gut microbiome. Therefore, the functional alterations of the gut microbiome induced by Cd exposure in *C. longicaudatus* were further explored. In this study, significant differences were observed between the treatment groups and the control group in numerous gene pathways associated with various substances and energy metabolism (Figure 7). These findings are consistent with a previous investigation conducted on mice (He et al., 2020), suggesting that Cd exposure significantly impacts the metabolic function of gut microbiota in *C. longicaudatus*. Significant increases in metabolic pathways associated with disease and environmental processing in the gut microbiota suggest that Cd ingestion promotes disease development and enhances the adaptability of *C. longicaudatus* to adverse circumstances. Furthermore, our findings indicate a significant downregulated of the pathways associated with amino acids, carbohydrates, and energy following Cd exposure, which has been implicated in the pathogenesis of type 2 diabetes (T2DM) (Bashir et al., 2019; Sanchez‐Alcoholado et al., 2017; Tinkov et al., 2017). Hence, it can be inferred that the alterations observed in the gut microbiota metabolism of *C. longicaudatus* in this study may contribute to the development of Cd‐induced T2DM. Notably, the metabolic functions associated with cysteine and methionine exhibited significant decreases, whereas the repair system and phosphotransferase system demonstrated significant increases, verifying the pivotal role of cysteine in the endogenous detoxification mechanism and the induction of metal exposure in vivo (Quig, 1998). The recent studies have demonstrated that methionine plays a crucial role in regulating metabolic processes, the innate immune system, and digestive functions in mammals. It also interferes with lipid metabolism, activates endogenous antioxidant enzymes, and promotes glutathione biosynthesis to counteract oxidative stress (Martínez et al., 2017). Glutathione serves as a vital constituent of the endogenous antioxidant defense system, and the imbalance in antioxidants has been linked to Cd toxicity (Zhao, Nyman, & Jönsson, 2006). Therefore, the results of this study suggest that *C. longicaudatus* can effectively mitigate Cd toxicity invasion by regulating its own metabolic function and related repair system. In conclusion, exposure to Cd induces significant alterations in both the composition and function of gut microbiota in *C. longicaudatus*, which serves as an innate defense mechanism against external harm.

## 5 CONCLUSION

This study demonstrates that Cd exposure can induce liver, spleen, and kidney damage in *C. longicaudatus*. It also alters gut morphology, potentially increases gut permeability and inflammation, contributing to the phenotypic changes. Specifically, the gut microbiota regulates itself to resist the damage caused by Cd exposure, resulting in an increase in potential pathogenic bacteria and a decrease in beneficial bacteria in the gut. The disruption of gut microbiota was associated with significant metabolic alterations, characterized by a notable decrease in over half of the metabolic functions and a substantial increase in pathways related to disease and environmental information processing in the Cd‐treated group compared to the control group. These data suggest that exposure to Cd has detrimental effects on both the structure and function of the gut barrier as well as gut microbes. This study can be used as a new approach to comprehend the stress response of wild mammals to Cd exposure, and to further assess the significant impact of Cd pollution on ecosystems by investigating the structural and functional changes in their digestive system and the disturbance of gut microbiota.

## AUTHOR CONTRIBUTIONS

**Mengfan Tao:** Data curation (lead); formal analysis (lead); investigation (lead); validation (lead); visualization (lead); writing – original draft (lead); writing – review and editing (lead). **Kanglin Cao:** Investigation (equal); resources (equal). **Xinsheng Pu:** Investigation (equal); resources (equal). **Yu Hou:** Investigation (equal); validation (supporting). **Lei He:** Investigation (equal). **Wei Liu:** Investigation (equal). **Yue Ren:** Data curation (equal); funding acquisition (supporting); supervision (equal). **Xin'gen Yang:** Conceptualization (lead); data curation (equal); funding acquisition (lead); supervision (lead); writing – review and editing (equal).

## FUNDING INFORMATION

The Basic Research Program of Shanxi Province, China (202303021221093, 202303021212109), Excellent Doctoral Award of Shanxi Province for Scientific Research (SXBYKY2022123), and the Grant from Shanxi Agricultural University (2023BQ46, 2023BQ47, ZBXY23B‐14) support for this work.

## CONFLICT OF INTEREST STATEMENT

The authors declare no competing financial interests.

## Data Availability

I confirm that the Data Availability Statement is included in the main file of my submission and that access to all necessary data files is provided to editors and reviewers. doi:10.5061/dryad.zgmsbcckt
